# Osteopathic Manipulative Treatment and the Management of Headaches: A Scoping Review

**DOI:** 10.7759/cureus.27830

**Published:** 2022-08-09

**Authors:** Cesar E Jara Silva, Andrew M Joseph, Mohammed Khatib, Jenna Knafo, Monica Karas, Kristina Krupa, Benjamin Rivera, Alexander Macia, Bhargavi Madhu, Mary McMillan, Jason Burtch, Jonathan Quinonez, Trevine Albert, Deepesh Khanna

**Affiliations:** 1 Foundational Sciences, Dr. Kiran C. Patel College of Osteopathic Medicine, Nova Southeastern University, Fort Lauderdale, USA; 2 Foundational Sciences, Dr. Kiran C. Patel College of Osteopathic Medicine, Nova Southeastern University, Clearwater, USA; 3 Osteopathic Medicine, Nova Southeastern University Dr. Kiran C. Patel College of Osteopathic Medicine, Fort Lauderdale, USA; 4 Osteopathic Neuromusculoskeletal Medicine, Larkin Community Hospital, Miami, USA; 5 Interventional Pain, Larkin Community Hospital, Miami, USA; 6 Foundational Sciences, Nova Southeastern University Dr. Kiran C. Patel College of Osteopathic Medicine, Clearwater, USA

**Keywords:** tth, headache, omt, trigger point, myofascial release, craniosacral, management of headaches, tension-type headaches, migraine headaches, osteopathic manipulative treatment (omt)

## Abstract

Headaches have been studied and treated since nearly 7,000 BC because of their significant global impact. Current headache treatment modalities are various and have a wide variety of targets, but medications are the most common. Since conventional medical treatments have several side effects, alternative remedies such as osteopathic manipulative treatment (OMT) should be considered. OMT can assist in the management of various health conditions, such as low back pain, neck pain, and headaches. The purpose of this scoping review is to evaluate recent findings regarding the efficacy of OMT modalities in the management of headaches such as tension-type headaches (TTH) and migraines.

This study was designed as a scoping review to gather evidence on the efficacy of OMT modalities in the management of headaches. Following PRISMA guidelines, four databases were used to search for articles published between 2010 and 2022 that reported the use of OMT and manual therapy for TTH and migraines. Databases used include Embase, PubMed, Medline, and Web of Science. The following keywords were used: treatment, therapy, Headache, migraine, craniosacral, muscle energy, myofascial release, trigger point, osteopathic, and manipulation. The initial search yielded 473 unique articles after removing duplicates. After screening based on the inclusion and exclusion criteria, and after further analysis, 15 articles were selected.

Data reports of OMT and manual therapy efficacy and/or effectiveness in treating TTH and migraine were analyzed. Articles included were randomized control studies (13 of 15, 86.6%), one pilot study (one of 15, 6.7%), and one case series (one of 15, 6.7%), which were divided into TTH (nine of 15, 60%) and Migraine Headaches (six of 15, 40%). All articles reported significant headache improvement in at least one measurement. Of all treatments analyzed, single technique interventions (seven of 15, 47%) and multiple technique interventions (eight of 15, 53%) were identified. Among the techniques used, Myofascial Release was the most common (nine of 15, 60%).

The articles presented provide evidence of the significant benefits of manual therapy. Because of the limitations of traditional medicine, OMT can be used either as an alternative or adjuvant therapy for headaches. Evidence suggests the positive impact it can provide on headache management, but the number of randomized control trials and population samples should be increased to support its recommendation. This demonstrates how different osteopathic techniques can provide therapeutic effects on TTH, MH, and potentially other types of headaches. A preference for myofascial release was observed, which can be due to the fast relief from the physiologic effect on tissue movement.

This review study demonstrates the benefits OMT has on decreasing headache frequency, intensity, and duration in TTH and migraines. OMT has shown to be beneficial, especially for patients seeking alternative non-pharmaceutical and non-invasive treatments. Further studies are needed to evaluate the effects of different OMT techniques, and different combinations of treatments, on other types of headaches.

## Introduction and background

Headaches have been one of the most common and prevalent medical concerns affecting the human population worldwide [1]. The prevalence of active headache disorder is an astounding 52.0%, affecting nearly one in every six Americans [2,3]. As such, headaches are the fourth leading cause of emergency department visits in the US and among the top 10 causes of disability worldwide [3,4]. Not only do headaches have physical symptoms, but also carry a financial burden of nearly 14 billion dollars per year in the United States [5]. Because of their significant impact on the global scale, headaches have been studied and treated since nearly 7,000 BC [6]. Headache pathology can be classified into two categories: primary and secondary headache disorders [7,8]. Primary headaches are the most common and referred to as symptoms arising directly from pathological discrepancies in the head [7]. Within this category, the two most common types are tension-type headaches (TTH) and Migraine Headaches (MH), each with a global prevalence of 40% and 10%, respectively [7]. On the contrary, secondary headaches arise as a symptom of an underlying disease such as acute sinusitis, brain aneurysm, and meningitis [5,7].

TTH is the most common headache type worldwide and has an etiology that arises from a constellation of emotional, environmental, and physical factors [9]. It has an onset between the second and third decade of life and is most prevalent between the third and fourth decades [10]. While the cause of TTH has not been conclusively identified, evidence suggests a major contributory role of pericranial myofascial tissue nociception along with increased central nervous system excitability [10]. Duration of TTH can last between 30 minutes to seven days and is diagnosed when two of the following four characteristics are present: 1) bilateral, 2) pressing or tightening quality without pulsation, 3) mild to moderate intensity, and 4) not exacerbated by routine physical activity [7].

Also classified as a primary headache, MH is the second most common type of headache which carries a greater physical burden on the population [3]. MH appears most commonly between 25 and 55 years and is three times more prevalent in females [5]. In MH, sufferers can experience the headache with or without aura, and focal neurologic symptoms, including visual disruptions, unilateral paresthesia, and language disturbances [7]. The pathophysiology is complex, attributing to abnormal cortical activity or cortical spreading depression and abnormal brain stem activity in the population with aura [10]. Furthermore, the pain originates in the sensory fibers that transmit pain signals from intracranial and extra-cranial blood vessels [10]. Treatment of these disorders is targeted through various approaches, but conventional therapy with medical care is the primary [5].

As one of the most common disorders, headaches have a wide variety of medications in an extensive and diverse pharmacology market [11]. The MH pharmaceutical industry was valued at $1.71 billion in 2017 and is expected to reach $2.2 billion by 2025 [11]. Remedies such as nonsteroidal anti-inflammatory drugs (NSAIDs), acetaminophen, and triptans all rapidly alleviate symptoms, but come with a myriad of side effects ranging from kidney and liver damage, ulcers, and even risk of ischemic vascular events [12]. Additionally, certain populations including children, elders, and pregnant women are at an even greater risk of experiencing adverse drug side effects [12,13]. Elderly patients are particularly susceptible to adverse drug reactions due to multiple medications and comorbidities, cognitive and functional impairment, and age-related changes in pharmacokinetics and pharmacodynamics [14]. Because of the repercussions of conventional treatment, alternative remedies like acupuncture and medical marijuana have been considered [15]. Patients have an overall favorable view of using medical cannabis, as it was reported to decrease the frequency and duration of migraines [15]. Among the alternatives, an integrative and structured therapy to take into consideration is osteopathic manipulative treatment (OMT) [16]. As a treatment modality, OMT can assist in the management of various health conditions, especially for disorders such as low back pain, neck pain, and headaches [16].

OMT is a branch of medicine that follows four tenets: the unity of the human body, the body's ability to regulate and heal itself, the interrelationship of structure and function, and the use of rational treatment revolves around the total care of the patient [17]. OMT includes a variety of procedures using the hands in the diagnosis and treatment of patients, which involve the body framework - ligaments, muscles, fascia, tendons, and joints, as well as the vascular and neurological aspects involved with these structures [17]. OMT treatment can be classified into four different models which determine the types of techniques for specific issues such as biomechanical, neurologic, respiratory/circulatory, and psycho-behavioral models [17]. Concerning headaches, the biomechanical, neurologic, and respiratory/circulatory models target the pathophysiology of the disease [17].

The techniques used in these models include myofascial release (MFR), muscle energy, high velocity and low amplitude (HVLA), trigger point, balanced ligamentous tension (BLT), occipito-atlantal decompression (OA-D), and cranial therapy treatment. MFR techniques are specific maneuvers directed toward the body’s soft tissues, principally the muscles and fasciae [17]. Evidence shows that MFR treatment in 10 weeks significantly improved a variety of different body functions such as basal metabolism, diastolic blood pressure, pain, and quality of life [18]. The muscle energy technique (MET) follows contraction and relaxation methods to solve or improve somatic dysfunctions [17]. MET produces a significant decrease in corticospinal and spinal reflex excitability, suggesting an overall decreased motor excitability [19]. OA-D consists of steady pressure applied to the suboccipital area to relax and normalize reflex action, resetting parasympathetic activity [17]. OA-D improves blood flow to the brain, which is explained by either parasympathetic stimulation through the secretion of vasodilating neurotransmitters or decreased external tissue pressure on the internal carotid artery and vertebral artery [20]. Furthermore, OA-D activates the parasympathetic anti-inflammatory reflex and cardiac parasympathetic tone and provides an antihypertensive effect, which may be mediated by reduced sympathetic modulation of vascular tone and/or increased baroreceptor reflex sensitivity [21,22].

Combining these techniques can provide significant effects on the treatment of diverse conditions [22]. For instance, OMT effectively increased brachial blood flow and stimulated the vagal system in patients with heart failure [23]. Similarly, there are indications that the physiological effects of OMT can contribute to the relief of headaches [24]. Most OMT techniques improve the flow of the lymphatics system as well, whereas soft tissue movement directly promotes lymph node drainage [17]. Recent findings by Absinta et al.'s results showed the presence of lymphatic vessels on human and nonhuman primate meninges, which can further contribute to another possible target for OMT on headaches [24]. Ultimately, OMT provides a vast array of treatment modalities to address headache treatments. Therefore, the purpose of this study was to identify how efficacious OMT is for TTH and MH treatment, and if OMT provides a substantial relief or resolution of associated symptoms.

## Review

Methods

A computerized search was performed to identify the efficacy of OMT treatment for TTH and MH. The databases PubMed, Embase, MEDLINE, and Web of Science were utilized with search terms related to OMT, headaches, TTH, and migraines.

Search strategy

Firstly, the inclusion and exclusion criteria were established before conducting the review. Articles were included if they (1) were in the English language, (2) were published from 2010 to 2022, and (3) included an abstract containing the keywords treatment/therapy, osteopathy/osteopathic manipulation treatment, or headache/migraine. Book review, print media, editorial, correspondence, short survey, erratum, conference abstract, and paper were excluded.

Identification of studies

An electronic search of PubMed, Embase, MEDLINE, and Web of Science was performed to identify effectiveness of OMT for TTH and migraines. An initial Boolean and keyword search that included "(("Crani"*" AND ""Sacral"") OR "("Muscle"" AND ""Energy"") OR "("Myofascial"" AND ""Release"") OR "("Trigger"" AND ""Point"") OR "("Craniosacral"" OR ""Cranio-sacral"") OR "("Osteopath"*" AND ""Manipulat"*")) AND "("treatment"" OR ""therapy"") AND "("headache"" OR ""migraine"") was conducted.

Data extraction

**Figure 1:**
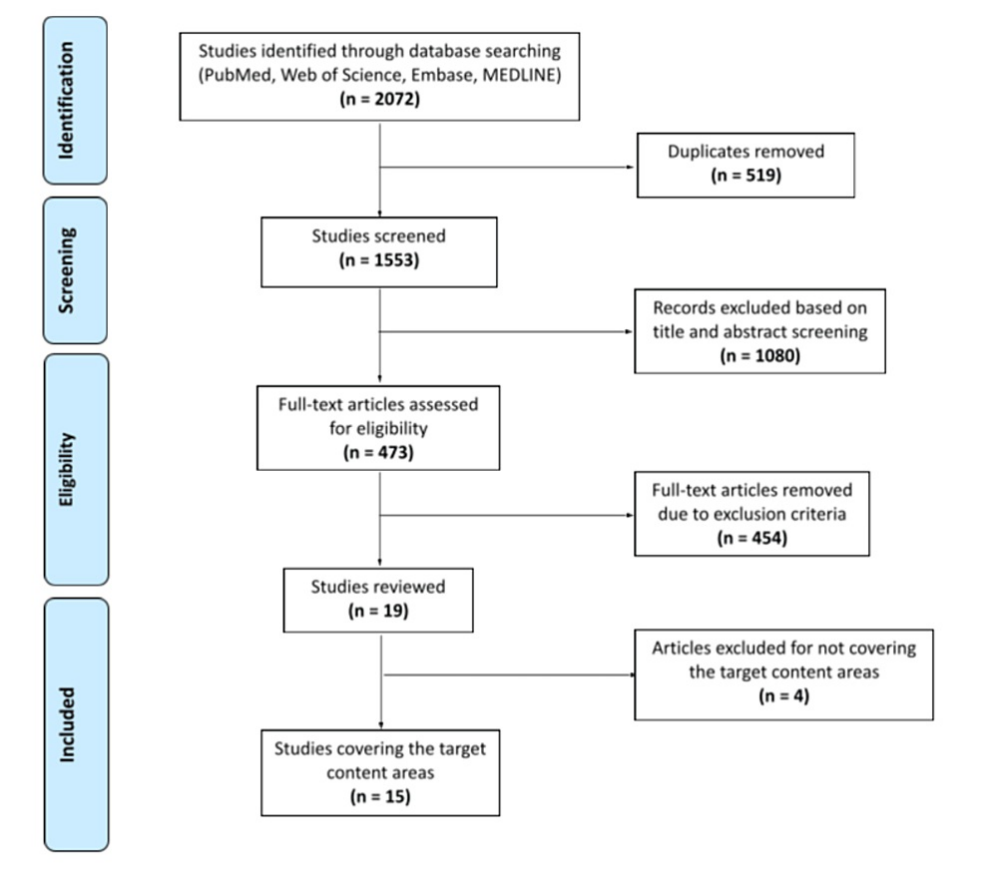
PRISMA flow diagram PRISMA: Preferred Reporting Items for Systematic Reviews and Meta-Analyses

The Boolean and keyword search resulted in 2,072 articles, and 519 duplicates were removed. Of the 1,553 articles that remained, 1,080 articles were removed due to either (1) the abstract not including all keywords (treatment, therapy, OMT, manipulation, craniosacral, muscle energy, MFR, trigger point, and headache) or (2) being a book review, print media, editorial, correspondence, short survey, erratum, or conference abstract or paper. Additionally, any article not written in English was removed. Two readers assessed the remaining 473 full-text articles based on the inclusion and exclusion criteria, and a total of 19 articles were found to be eligible. Upon further screening, four did not include TTH or MH, leaving 15 articles for this review (see Figure 1).

Results

The search yielded a total of 2,072 articles using the predetermined search criteria. After 519 duplicates were removed, book reviews, correspondence, editorials, podcasts, radio, TV, newspaper, print media, letters, notes, conference abstracts, short surveys, erratum, conference papers, or chapters were excluded. An additional 1,080 articles were removed due to the language of publication not being in English, the abstract not including all the keywords “osteopathic manipulative treatment”, “migraine headache”, or “tension-type headache”. The remaining 473 full-text articles were then assessed based on the inclusion and exclusion criteria, and a total of 19 articles were found to be eligible. Upon further screening, 4 did not include TTH or MH, leaving 15 articles for the final analysis.

Table 1 reports the characteristics of the 15 studies included in the review, where most were randomized control studies (13 of 15, 86.6%), one pilot study (one of 15, 6.7%), and one case series (one of 15, 6.7 %), which targeted the effects of manual manipulation in the treatment of headaches. Among the reviews, two specific headaches were primarily focused on TTH (nine of 15, 60%) and MH (six of 15, 40%). The articles were analyzed in Table 1, where the factors contributing to the results were evaluated. Furthermore, the specific technique treatment and its physiologic effect on each type of headache were deeply considered.

**Table 1:** Studies on different OMM techniques on migraine and tension-type headaches

Study Citation Details	Purpose of Study	OMM Techniques Used	Measures	Key Findings
Migraines Headache				
Arnadottir et al. (2013) [25]	To assess the effectiveness of craniosacral therapies in the alleviation of symptoms in migraines	Myofascial tissue release, soft tissue techniques, as well as light touch handling.	Short-Form Headache Impact Test (HIT-6) Questionnaire	The total HIT-6 scores showed a significant difference when utilizing Wilcoxon’s t-test to analyze scores before treatment (I) and right after treatment (II) (t 1⁄4 2.37, p 1⁄4 0.018) with an effect size (ES) of 0.48. The total HIT-6 scores before treatment (I) and one month after treatment (III) (t 1⁄4 2.09, p 1⁄4 0.037) showed a significant change, with an effect size of 0.43. A statistically significant difference (t 1⁄4 2.91, p 1⁄4 0.004) was found with Wilcoxon’s t-test of the mean HIT-6 scores between groups at the start of the research (Time 1 1⁄4 61) and at the conclusion of the research (Time 4 1⁄4 55) with an effect size of 0.55.
Cerritelli et al. (2015) [26]	To assess the effectiveness of osteopathic modalities in the treatment of chronic migraines	Balanced membranous and ligamentous tension, direct and indirect myofascial release, cranio-sacrum	HIT-6 questionnaire, functional disability, drug consumption, migraine pain intensity, and frequency.	OMT groups were statistically different from the control (p < .001) and sham group (p < .001) amongst the three samples (p < .001). OMT groups had a statistically significant reduction in all factors when compared to control (p < .001) and sham (p < .001) as shown by the Tukey post hoc analysis. Migraine frequency per month differed significantly among the three groups at the end of the study period (p < .001). Also, OMT groups showed a significantly decreased migraine frequency compared to control (p < .001) and sham (p < .001) groups. The control group was significantly different from the sham group (p < .001).
Gandolfi et al. (2018) [27]	To evaluate myofascial and trigger point treatment effectiveness in chronic migraine patients taking prophylactic treatment with onabotulinumtoxinA.	Myofascial release and trigger point	HIT-6, the Migraine Disability Assessment Scale (MIDAS), cervical active range of motion with CROM3 goniometer, and pressure pain threshold (PPT) with a Wagner algometer	When patients’ status-post treatment was assessed, analgesics and NSAIDS total consumption were significantly lower with osteopathic manipulative treatment compared to transcutaneous electrical nerve stimulation (TENS) treatment (P=.02 and P=.02, respectively). The threshold values of pressure and pain in the upper trapezius (P=.02), occipital (P=.004), and temporal (P=.002) muscles were significantly lower with osteopathic manipulative treatment than with TENS. The total consumption of analgesics (P=.009), NSAIDs (P=.01), and triptans (P=.015) in the patients was significantly lower after treatment with osteopathic manipulation when compared to before the intervention. These findings thus revealed significant improvements in cervical active range of motion and trigger point sensitivity.
Espí-López et al.; (2018) [28]	To determine the efficacy of suboccipital inhibitory techniques in patients with migraines	Myofascial trigger point and suboccipital soft tissue inhibition	HIT-6, quality of life by the Short Form Health Survey (SF-36), and disability by the Migraine Disability Assessment (MIDAS)	Both groups receiving myofascial trigger point therapies and suboccipital soft tissue inhibition were found to have a significant reduction with statistical analysis of HIT-6 score for impact of headache (p=.001)
Voigt et al. (2011) [29]	To compare the efficacy of OMT in females with migraines to traditionally used medical therapies	Manual, visceral, or cranial techniques	Migraine disability assessment and SF-36, PAIN questionnaire, and healthcare quality of life (HRQoL).	Female migraine patients treated with OMT showed statistically significant improvements in pain, HRQoL, and working disability. The intervention group showed a great decrease in pain intensity (p < .05) from t1 to t2 and from 66.7 to 53.8 (on a scale of 0 for no pain to 100 for worst imaginable pain). The PAIN questionnaire scores were also reduced from 70.8 to 51.5 on an equal scale. When assessing Working Disability, there was a significant (p < .05) decrease in disturbance of occupation due to migraines, as seen in the ‘‘Pain Questionnaire’’ in the intervention group (66.7–50.0, on a negatively polled scale from 0 to 100). In the MIDAS questionnaire, a significant decrease (p < .04) in disablement days (2.5 to 0.5 days) was also shown. In t1, a total of 19 subjects admitted their migraines had impacted their occupations, which reduced to a total amount of 17 in t2.
Muñoz-Gómez et al. (2022) [30]	To assess the efficacy of craniosacral therapy in migraine patients, compared to a placebo treatment.	Frontal technique, Suboccipital inhibition technique, fourth ventricle technique, lumbosacral technique, and sphenoid technique	Migraine pain intensity, severity, episode frequency, functional, emotional, and overall disability, medication intake, and perceived change following treatment	Upon analysis of pre and post-intervention groups, a reduction of patients experiencing severe migraine pain (64 to 24%) was seen. For individuals’ post-treatment, a reduction in the functional and overall disabilities was seen at 23.21% and 23.02% respectively. Upon analysis of post-intervention follow up these measures were seen to be at 21.12%. When compared to sham groups, patients receiving craniosacral therapy were found to have significantly reduced functional and overall disability (p=.001 and p=.02 respectively), episode frequency (p = .001) with a significant reduction in pain (p=.01). 52% of patients who received craniosacral therapy reported improvement following treatment at the post-interventional stage with a higher self-reported perception of change (p-.01), and a reduction in their medication usage by 36.04% and 31% during post-intervention, and post-intervention follow up respectively even when compared to sham groups (p=0.01).
Tension Type Headaches				
Ajimsha (2011) [31]	To assess the effectiveness of direct and indirect MFR in treating tension headaches	Indirect and direct myofascial release	Headache frequency after MFR	The number of headaches reported in days within a 4-week period, or Headache frequency, resulted in a decrease by 7.1 (SD - 2.6, direct MFR) compared to 6.7 (SD – 1.8, indirect MFR) and 1.6 (SD – 0.5, Control).
Corum et al. (2021) [32]	To identify differences in efficacy of various osteopathic treatment techniques for headache symptom relief	Indirect and direct myofascial release, high velocity, and low amplitude (HVLA) techniques	Headache frequency, duration, and intensity. HIT-6, Neck disability Index (NDI), Pressure Pain Threshold (PPH) at immediate post-treatment, and at 3 months after	Headache frequency was significantly reduced when measured after treatment (− 3.3 ± 1.2; p = .002) and at follow-up in three months (− 3.0 ± 2.1; p = .003) in the manipulation group. The post-treatment headache frequency in the manipulation group showed a significant difference compared to the control group (p < .001). HIT-6 scores showed a significant decrease in the manipulation group when measured after treatment (p=0.002) and at follow up in three months (p=.041). Intensity of neck pain showed a significant reduction in the manipulation group (p=.007) and in the myofascial release group after treatment (p = .009).
Deodato et al. (2019) [33]	To assess effectiveness of osteopathic techniques on tension type headaches	Myofascial release (MFR), Muscle energy, articulatory techniques, balanced membranous tension, and cranial techniques	Photogrammetry and Radiography of craniovertebral angle. The cephalic outcome, involves frequency, intensity, and duration of headache.	Statistically significant changes in headache measurements were seen in OMT patients: pain intensity showed a decrease from a mean score of 4.9 (SD = 1.4) to a mean score of 3.1 (SD = 1.1) (P=.002); frequency was also shown to decrease from 19.8 days (SD = 6) to 8.3 (SD = 6.2) days per month (P=.002), and duration of headache also shown to decrease from 10 hours (SD = 4.2) to 6 (SD = 3) hours (P=0.01). In the control group, significant improvement was found in pain intensity, which improved from a mean score of 5.9 to 4.2 (P=.03); frequency was reduced from 23.4 to 7.4 days per month (P=.003), and duration diminished from 7.8 to 3.6 hours (P=.002). Forward head posture in OMT patients also improved significantly (P=.003).
Mohamadi et al. (2020) [34]	Investigation of positional release techniques (PRT) and its effects on central sensitization in populations with chronic TTH	Positional release techniques	Brain metabolite profiles as primary outcome measured. Secondary measured outcomes consisted of headache intensity and frequency, McGill score, McGill Pain Questionnaire, self-reports, pressure algometer as well as pressure pain threshold (PPT.)	Group comparisons of the PRT group after treatment revealed a significant decrease in headache frequency (p=.001), intensity (p=.002), and McGill score (p=.003), with a significant increase in pain threshold (p=.003). On the other hand, no significant changes in metabolite profiles were found after treatment. Within control groups, an increased M-INO/Cr ratio was found within the somatosensory cortex (p=.041). When compared to the control group, PRT group revealed significant differences in headache frequency (p=.001)
Cho (2021) [35]	The validation of the positive impact suboccipital myofascial release (MFR) techniques and forward head posture (FHP) correction exercises have in treatment of chronic TTH	Therapies consisted of FHP correction exercises, suboccipital muscle inhibition (SMI), and MFR	Measured outcomes included the headache pressure pain threshold (PPT), HIT-6), soft tissue myofascial trigger points, postural kyphosis, and FHP angle	SMI and FHP techniques showed significant improvements in posture, HIT-6, trigger points, soft tissue PPT, and headache PPT. Within the combined SMI technique and FHP correction exercises group, there was the largest reduction of headache PPT and HIT-6.
Moraska (2015) [36]	To examine the efficacy of headache pain reduction using trigger point release massage (TRP) directed towards myofascial trigger points (MTrPs)	TPR focused on MTrPs	Headache frequency, pain, duration and intensity, and perceived difference in pain during headache, and Pressure-pain threshold (PPT)	Differences in group treatments identified changes in frequency of headaches over time (F (6, 52) = 2.65, p=.026). On post-hoc analysis, headache frequency decreased in both placebo (p=0.013) and massage (p=.0003) compared to their baseline. A significant decrease was observed in HDI scores for the treatment group (p = .0003) but not in the placebo (p = .06) or wait-list (p = .39) groups; a significant change was found in HIT-6 scores over time in both the treatment (p = .0002) and placebo (p = .011).
Espí-López et al. (2014) [37]	Investigation into two types of therapies aimed at the suboccipital region to determine effectiveness for management of tension-type headaches.	Manual therapies consisted of occiput-atlas-axis global manipulation and suboccipital soft tissue inhibition as well as a combination of both techniques	Outcomes were recorded as measures of impact, disability, pain, and intensity of headaches as well as a headache diary and range of motion of craniocervical junction	Significant improvements were seen at the 8 weeks post-treatment follow-up in comparison to the pre-intervention, where 66.7% of participants reported suffering from headaches described as moderate intensity, and a rating of 6.49 with a standard deviation of 1.69 in the level of their average pain.
Ghanbari et al. (2012) [38]	Comparison of effectiveness of trigger point management by positional release therapy to routine medical therapy for TTH	Indirect PRT	Daily headache diary and pressure algometry at trigger points	During the comparison between the study groups, a significant change was not found in frequency of headache (P= .508), intensity (P= .064), duration (P= .486), and tablet count (P= .783), and no significant reduction was found in headache intensity within the PRT or medication groups. After the treatment phase in both PRT and medication groups, there was a significant improvement in headache frequency, duration, and tablet count.
Choi (2016) [39]	To analyze the effects of cervical traction treatment compared to the McKenzie exercises on patients with neck muscle stiffness associated with tension-type headaches	Cervical traction, McKenzie exercises, and cranial rhythmic impulse	Muscle tone and tension	Headache frequency decreased in the cervical traction group (p < .05) but no statistical difference was found in the cranial rhythmic or McKenzie exercise groups.

Migraine headaches

Techniques Utilized

Various OMT techniques were assessed across six studies to evaluate their impact on MH. Myofascial tissue release, both direct and indirect, was the most frequently examined OMT technique in the treatment of MH, being used in three out of six studies [25-27]. The myofascial trigger point technique was also evaluated in two studies [27,28]. Cranial OMT and its effect on treating MH were studied in 33% of the six studies [26,29]. The most common soft tissue technique tested during OMT treatments was suboccipital inhibition [28,30]. Additional soft tissue techniques examined include frontal technique, sphenoid technique, fourth ventricle technique, and lumbosacral technique [30]. One study evaluated the impact of the OMT techniques BLT and balanced membranous tension (BMT) on MH [26].

Measuring Instruments

A variety of measurement instruments were used across 6 OMT studies to evaluate and quantify the effectiveness of various OMT techniques in the treatment of MH. The most commonly used measurement tool across the 6 studies was the Headache Impact Test-6 (HIT-6), which was used in 4 out of 6 studies [25-28]. The Migraine Disability Assessment Scale was used in 50% of the studies that evaluated OMT and MH [27-29]. Two studies incorporated the use of a standardized migraine diary [26,30]. Other measurement tools used to measure the effectiveness of the various OMT techniques in the treatment of migraines include a Short-Form Health Survey (SF-36), Visual Analog Scale, Headache Disability Index, cervical active range of motion with CROM3 goniometer, and pressure pain threshold with a Wagner algometer [27,28,30].

Control and Comparison Groups

All studies in this review utilized a control or sham group except a study by Arnadottir et al., who utilized an experimental crossover design [25]. Two of the studies utilized a sham group by providing hands-on maneuvers to control statistical variabilities within their study [26,30]. Remaining studies utilized different treatment modalities for control groups, with their experimental group having the addition of physical treatment to supplement. These studies’ control groups consisted of established treatments or alternate supplemental procedures such as transcutaneous electrical nerve stimulation (TENS) therapy [27].

Study Population

The majority of studies included in this analysis included both male and female research subjects and excluded participants under the age of 18 years old and over the age of 65 years old. One study excluded individuals under 20 years old, including an age range from 20 to 50 years old [25]. All other studies included subjects that were over the age of 18 [26-29]. Two studies evaluated subjects ranging from 18 to 60 years old, while Another study included subjects up to 50 years old [26,28,30]. Two studies included patient populations between 18 and 65 years old, and one study only included female patients [27,29]. While inclusion criteria varied based on the number of migraines per month, the most commonly used criteria were the International Classification of Headache Disorders (ICHD) criteria. The majority of studies took place within the European continent with two in Italy, two in Spain, one in Germany, and one in Iceland.

Length of Treatment Session and Length of Overall Study

The studies included in this analysis varied in their treatment protocol and the total length, ranging from as little as four weeks to as long as six months. One study lasted four weeks, consisting of Myofascial and trigger point treatment sessions that lasted 30 minutes and occurred once a week [27]. Another study lasted eight weeks, with one treatment session per week, which included suboccipital inhibition techniques that lasted 10 minutes each [30]. Another study lasted 12 weeks with six treatment sessions per month consisting of Myofascial Tissue Release and Soft Tissue Techniques [25]. Three studies took place over six months but varied in their treatment protocols [26,28,29]. Of these studies, one study included eight treatment sessions with MFR and BLT [26]. Another study spread its therapeutic intervention over eight weeks (with treatment occurring every 15 days), with a treatment consisting of Myofascial Trigger Point plus Suboccipital Soft Tissue inhibition, lasting 30 minutes per session in the experimental group and 20 minutes per session in the control group [28]. The final study included five 50-minute sessions of manual, visceral, or cranial OMT on female patients [29].

Statistical Results

The studies assessing the impact of treatment with migraines used various criteria for quantification of results, such as the Headache Impact Test -6, Migraine Disability Assessment (MIDAS), or Headache Disability Index (HDI) [28-30]. These validated measures were used in isolation, conjunction, or combination with other measures to quantify patient experience and perform statistical analysis. All selected literature revealed a notable significant difference in treatment groups, including decreased measures of migraine severity or pain. One notable exception is Gandolfi et al. who found no significant difference in headache-related variables in populations receiving myofascial and trigger point treatments, although a significant decrease in acute medication use after manipulative treatment was found [27]. In studies assessing HIT-6 scores, OMT reduced the score by 8 (95% CI: −12.96; −4.52), Craniosacral therapy by 7 with a CI of -11.5; -2.68, and craniosacral therapy by 6 with a standard deviation of 10.5 [25,26,28].

Tension-Type Headaches

Techniques Utilized

Osteopathic Manipulative Techniques (OMT) were used across nine studies to treat TTH. Of the OMT techniques used, three out of nine used MFR to treat TTH [31-33]. Two of the studies used purely positional release techniques (indirect) to study the effect of OMT on TTH [34,35]. while another study used a combination of cervical traction and McKenzie exercise [36]. One study combined the Osteopathic techniques of suboccipital soft tissue inhibition, and occiput-atlas axis global manipulation [37]. Another study only used myofascial trigger point-focused massage techniques for recurrent TTH [38].

Measuring Instruments

A variety of instruments were used to measure the effectiveness of various OMT techniques in these studies. Most studies used multiple means of measuring treatment effectiveness. The most commonly used instrument for measuring treatment effectiveness was a headache diary, used by four out of nine studies on tension headaches [31,33,36,38]. The second most common instrument was the Visual Analogue Scale (VAS), used by three studies [32,37,39]. The Pressure-Pain Threshold (PPT) was used by Mohamadi et al. and Moraska et al. [34,36]. The last instrument that was seen in multiple studies was the Headache Impact Test, or HIT-6, used by Corum et al. and Espí-López et al. [32,37]. Additional methods used individually to measure the effectiveness of interventions were the Myoton PRO (muscle tone measurer) by use of a digital force gauge, a pressure algometer, a self-report of perceived clinical change in headache pain, and pressure pain threshold (PPT) [34,36,38,39]. Numeric pain intensity, Headache Disability Inventory (HDI), brain metabolite profile, proton magnetic resonance spectroscopy, patients’ self-reports, and the McGill Pain Questionnaire were also used [34,37,38]. Additionally, Neck Disability Index and Cervical ROM measured with a CROM device by Espí-López et al. were also used [32,37]. Lastly, the Postural Assessment Software/Software for Postural Evaluation (PAS/SAPO) was used by Deodato et al. [33].

Control and Comparison Groups

Seven out of nine of our studies evaluating the use of osteopathic manipulation for TTH were randomized controlled trials (RCTs). One was a pilot study and one was a case series [33,39]. Of the seven RCTs, various controls were utilized. For two of the RCTs, soft stroking in the same location of MFR was performed as a control [31,35]. The other five RCTs had the following controls: a placebo of a detuned ultrasound, exercise, NSAIDs or triptans, and ibuprofen 200mg [32,36,38]. Other studies had no treatment, but the attendance of the same sessions as the interventional group [37]. The pilot study utilized amitriptyline of 30mg or 50mg based on body weight as the control [33].

Study Population

Our study populations included both male and female sex with varying age cut-offs depending on the study. Two studies were limited to ages 18 to 50 [31,35]. One study was limited to ages 40 to 49 years [39]. One study was limited to ages 18 to 59, and another from 18 to 65 years [36,37]. One study was limited to ages 19 to 48, while another study evaluated patients aged 20 to 40 years old [32,38]. Two of our studies had no age cut-off, but rather a minimum age of either 18 in one study or 25 in another study [33,34].

Inclusion criteria for the type of TTH and length of diagnosis varied based on the study. Inclusion criteria for the majority of the studies were based on the diagnosis of TTH utilizing the International Classification of Headache Disorders criteria for TTH [32-34,36-38]. Two of the studies utilizing this classification also required the presence of myofascial trigger points [34,36]. The inclusion criteria for two studies were diagnosis of episodic or chronic TTH lasting at least 12 months, and those who had completed a 4-week baseline headache diary [31,35]. One study had no limitations on time of diagnosis; instead, the inclusion criteria were that the patient complained of headache and tenderness in the cervical muscle and was diagnosed with infrequent/frequent episodic tension-type headache after treatment by a neurologist [39].

The studies included in this review took place worldwide. Two of the nine studies on TTH took place in Kerala, India [31,35]. Another two of the studies took place in Iran [34,38]. The other five studies took place in various locations such as Spain, Italy, Turkey, South Korea, and the United States [32,33,36,37,39].

Length of Treatment Session and Length of Overall Study

The studies varied widely in their total length, ranging from as little as five days to as long as 20 weeks. Only one study lasted five days, while one study lasted 4 weeks, one lasted five weeks, one lasted six weeks, three studies lasted 12 weeks, and one lasted 14 weeks [31-34,37,38].

The studies also varied in the length of each treatment session. One study utilized 20-minute sessions of cervical traction, cranial rhythmic impulse, and McKenzie exercises [39]. Another study utilized one-hour sessions of indirect and direct MFR through the use of slow soft stroking with the use of finger pads [31]. Another study utilized 45-minute sessions of trigger point release massages focused on myofascial trigger points in muscles most commonly associated with pain referral to the head, such as the upper trapezius, suboccipital, and sternocleidomastoid [36]. Another study customized treatments with an unspecified length using HVLA, OA decompression, and even exercise during treatments [32]. Another study utilized hour-long sessions of a variety of direct and indirect techniques, including muscle energy, articulatory techniques, MFR, balanced membranous tension, and cranial treatments, as well as incorporated the use of Amitriptyline treatments if necessary [33]. Another study with treatments of an unspecified length utilized indirect positional release therapy, as well as NSAID and triptan use if needed [38]. Another study customized sessions lasting 1-1.5 hours with positional release treatments and Ibuprofen if needed [34]. Another study utilized hour-long treatments of indirect and direct MFR [31]. Lastly, another study utilized treatments of varying lengths with ​​suboccipital soft tissue inhibition, occiput-atlas-axis global manipulation, and a combination of both techniques [37].

Statistical Results

All studies reviewed revealed statistically significant changes in headache parameters of patients before and after OMT, through their various criteria for measuring effectiveness in treatment. One study, however, showed that both positional release therapy and medical therapy are equally effective at treating TTH [33].

One particular study was able to present that direct and indirect MFR OMT treatments significantly decreased the number of TTH the studied population experienced within 20 weeks, with a confidence interval of 95% [31]. This study also compared the effectiveness of direct and indirect MFR at relieving TTH and interestingly found that patients treated with direct MFR had greater outcomes as the number of headaches per month decreased by 7.1 days in the direct MFR group, as compared to a decrease in 6.7 days in the indirect MFR group [31].

Another study looked at headache frequency, intensity, and pain threshold before and after intervention with positional release therapy, noting that headache frequency (p=.001), headache intensity (p=.002), and McGill score decreased significantly (p=.003) and local pressure pain threshold increased significantly (p=.003) after treatment [34]. Despite these significant findings, there were no significant changes in the metabolite profile of glutamate-glutamine/creatine in this group after treatment (p=.014) [34].

Discussion

The purpose of this study was to identify the efficacy of OMT on TTH and migraine headache treatment. The effectiveness of OMT was based upon the presence and quantity of relief of symptoms associated with TTH and MH.

*Migraine Headaches*

Main findings: OMT techniques such as direct and indirect myofascial tissue release were most commonly evaluated in the studies on MH [25-27]. Myofascial trigger point techniques, cranial OMT techniques, and soft tissue techniques such as suboccipital inhibition were also examined [26-30]. The Headache Impact Test - 6 (HIT-6) and Migraine Disability Assessment Scale were most commonly used to evaluate the effectiveness of OMT techniques on migraines. Most studies used hands-on maneuvers as part of their control groups, while a study by Arnadottir et al. used an experimental crossover design, and other studies used alternative supplemental procedures such as TENS therapy [25,27].

The patient population of these studies ranged from 18 to 65 years old, with one study evaluating specifically female patients, who are diagnosed with migraines as indicated by the International Classification of Headache Disorders (ICHD) criteria [29]. All studies were conducted in Europe.

Most studies in this review have shown a statistically significant difference in using OMT to alleviate migraines in patients. One study by Gandolfi et al., in particular, demonstrated no statistically significant difference in headache measurements in groups receiving myofascial and trigger point treatments, but a statistically significant decrease was noted in the use of relieving medications after OMT treatment when the study was conducted for four weeks [27]. On the other hand, other studies that demonstrated statistically significant decreases in headache frequency and intensity ranged from eight weeks in duration to six months. OMT techniques were specifically found to decrease pain intensity and improve mental health and daily living in female patients diagnosed with migraines [29].

Implications: The treatment of migraines through OMT techniques revealed significant results with improved patient outcomes. These outcomes were primarily based on the analysis of their validated quantifiable measures. It was found that OMT [26,29], craniosacral therapy, and soft tissue techniques were possible sources of treatment for migraines, impacting the duration and intensity of symptoms, drug consumption, or resulting functional disability [25,26,28-30]. A study by Gandolfi et al. evaluating the feasibility of myofascial and trigger point treatments found no significant difference in headache-related variables [27]. This might have been due to the short treatment sessions (30 minutes) that occurred only once a week for four weeks overall, which is the least period spent on treatment compared to the other studies included in this review [27]. Other studies that showed significant improvements ranged from eight weeks in duration to six months, indicating that at least eight weeks in duration is needed to see relief in the use of OMT on migraines. Additionally, groups receiving OMT treatment demonstrated a significant decrease in acute medication use after treatment, indicating that OMT techniques provide a temporary relief that is observed immediately post-treatment [27,30]. OMT techniques such as cranial techniques demonstrated significant improvements in quality of life when performed on female patients diagnosed with migraines, and also greatly decreased emotional disability in both gender populations [29,30].

Since the studies were conducted in Europe, the results can be generalized to the population in Europe, but further studies are needed to be conducted in the US to determine whether the conclusions can be generalized to the US population as well.

As the precise mechanism of action of the effect of OMT on migraines has not been defined, it has been hypothesized that it includes the rebalance of vegetative nervous system (VNS) nuclei and the reduction of pro-inflammatory substances [26]. Migraines were demonstrated to be associated with functional alteration of both VNS and specific autonomic nuclei responsible for pain perception and sustained pain. Similarly, Gandolfi et al. discussed the resulting decrease in proinflammatory substances when spinal manipulative treatment disrupted the pain-spasm cycle and therefore improved vascular circulation [27]. None of the studies were able to provide a direct/indirect measurement of possible physiological changes made by OMT. Furthermore, it should be taken into consideration the subjective perception of pain and discomfort from the patients as well as their perception of OMT. As Muñoz-Gómez et al. described, perception of change is important as it provides clinically relevant information on the perceived effect of treatment [30].

Overall, OMT has been shown to provide significant improvement in migraine management. A combination of OMT techniques, such as MFR and craniosacral techniques, can help reduce migraine intensity and frequency, and acute medication use, which can further contribute to the quality of life for patients suffering from migraines [26,27]. The utilization of these various OMT techniques can supplement current treatment modalities and provide more effective relief to patients suffering from migraines.

Tension-Type Headaches

Main findings: OMT techniques such as HVLA, MFR, positional release techniques, cervical traction, McKenzie exercise, suboccipital soft tissue inhibition, and Myofascial trigger point focused massage techniques were performed on patients with TTH to evaluate their efficacy. Headache diaries were most commonly used to measure the effectiveness of these techniques [31,33,36,38]. Other commonly used measurement techniques included the Visual Analogue Scale (VAS), Pressure-Pain Threshold, and Headache Impact Test, or HIT-6 [32,34,36,37,39]. Most studies were randomized controlled trials (RCTs) that used control groups such as soft stroking and a placebo of a detuned ultrasound [31,35,36].

The patient population of these studies ranged from 18 to 65 years old, with some studies limiting the age range to patients of either 20 to 40 years old or 40 to 49 years old [38,39]. Inclusion criteria for most studies included a diagnosis of episodic or chronic TTH that lasted at least 12 months and had completed a four-week long baseline headache diary before the study [31,35]. These studies took place in India, Iran, Europe, Turkey, South Korea, and the United States.

All studies in this review have shown statistically significant differences in their respective measurements regarding the effectiveness of OMT on TTH. Both positional release therapy and medical therapy were shown to be equally effective at relieving TTH, shown by the decrease measured in headache frequency and intensity [33]. Direct and Indirect MFR techniques were found to significantly decrease the number of episodes of TTH within 20 weeks [31]. Specifically, direct MFR techniques were able to decrease the number of headaches per month by 7.1 days, compared to 6.7 days by the indirect MFR techniques [31].

Implications: When reviewing the findings of each study regarding effectiveness in treating TTH, it can be deduced that intervention with OMT provided measurable instant relief of TTH in all scenarios. Relief was even found to be long-lasting, noting improvements in the patient’s posture over time as well, with treatments spread across a period [33]. Positional release therapy has shown to be effective at treating TTH by decreasing the headache frequency and intensity in patients [33]. Direct MFR techniques were found to be more effective compared to indirect myofascial techniques regarding decreasing the headache frequency in patients [31]. When comparing the effectiveness of different OMT techniques in treating TTH, it can be deduced that the most popular and effective treatments involve a combination of the following techniques: HVLA, positional release therapy, and direct MFR. With this in mind, customizable OMT treatment sessions for patients with TTH are a proven and highly recommended modality for improving the lives of patients who suffer from TTH.

Similarly, for TTH, there is no definite mechanism for the physiological effects of OMT. OMT techniques, including MFR, provide direct relaxation of muscle tension in cervical muscles and fascia, resulting in relief. Therefore, this can indicate that TTH causes dysfunction of pericranial myofascial tissue, which can explain part of the pathophysiology of TTH [31]. As Ajimsha et al. described, decreased fascia tissue length and elasticity caused by repetitive strain injury, physical trauma, and inflammation can be resolved with the MFR [31]. Pain relief can be secondary to the fascial tissue returning to its normal length [31]. Therefore, these mechanisms can contribute to the subjective sample response evaluated in the studies.

These findings can be generalized due to the moderate sample size in most studies, along with the use of standardized measurements such as HIT-6 and statistical analysis to ensure that the results are statistically significant. Additionally, the studies range from various countries across the world, allowing these results to be generalized worldwide.

Limitations of included studies

In attempts to include relevant studies through the database search, the possibility of missing clinically significant and relevant studies cannot be excluded. The use of four databases restricted the articles to be used. Limited translational resources restricted us from the use of articles that were not written in English. The inclusion criteria allowed for OMT performed in Europe and Asia to be included, where medical training is not as standardized compared in the United States. Similarly, there is inherent variability in techniques among different OMT practitioners and in including studies that used physical therapists instead of licensed OMT physicians. The inclusive use of randomized controlled trials limited the study to further analyze evidence in other experimental trials. Furthermore, the lack of quality evidence analysis limits the study for clinical recommendations. Other potential limitations of the studies in this review include low sample sizes, short duration of the study, and heterogeneity of the populations sampled.

Limitations of the review process

First, this study focused on articles published only in the past 10 years. This limitation does not allow for all published data on the topic of OMT treatment on TTH and migraines to be analyzed and covered in concluding. Additionally, this review only included articles written in English, which may not be representative of the global prevalence and treatment of TTH and MH. Lastly, relevant articles may have been excluded due to limited databases utilized and strict inclusion and exclusion criteria.

Future research considerations

Through this scoping review, we hope to provide supportive guidance for future research studies in OMT. One limitation of selected studies that has been mentioned is the lack of diversity within the patient populations. Some studies were able to work within the constraints of small population sizes by utilizing crossover studies [25]. Further use of crossover studies could allow researchers to assess the effects of multiple types of maneuvers within the same patient populations, creating a multimodal analysis of the different types of treatments available. Further recommendations relate to increasing the validity of results by avoiding possible confounding variables through the increased use of sham and control groups while using crossover groups, as well as using multiple standards and validated measures for patient populations.

Secondly, the database search identified a small number of articles executed in the United States. With the increasing rate of students graduating in the U.S. as Doctor of Osteopathic Medicine (D.O.), the unique effect of OMT should be further supported as management for diverse conditions. This review should be taken into account to expand the understanding and provide evidence of the benefits OMT can provide. Through the articles acquired, it can be observed how there are many hypotheses of OMT physiological effects and secondary pain relief effects. Further studies are needed to evaluate the mechanism of action of different OMT modalities on various conditions besides migraine and TTH headaches. Lastly, because of the lack of protocolized use of OMT in headache treatment, it will be significantly beneficial for a consensus to propose a standardized protocol of treatment.

## Conclusions

It can be concluded that OMT can be used through diverse techniques to provide significant improvements to headaches. Such benefits include decreasing headache frequency, intensity, and duration in TTH and migraines. The implementation of soft tissue techniques including MFR, suboccipital inhibition, and cranial therapies can not only assist in the management of migraine pain intensity and frequency but also in decreasing the use of medications. Furthermore, OMT has the potential to provide significant headache improvement as adjuvant therapy and has shown to be beneficial, especially for patients seeking alternative non-pharmaceutical and non-invasive treatments. Comparably, OMT techniques such as HVLA, positional release therapy, and MFR techniques can be used with significant efficiency for TTH management. Further studies are needed to evaluate the effects of different OMT techniques, and different combinations of treatments, on other types of headaches.
